# Anemia in Elderly Patients: Contribution of Renal Aging and Chronic Kidney Disease

**DOI:** 10.3390/geriatrics10020043

**Published:** 2025-03-14

**Authors:** Simone Santos, Irina Lousa, Márcia Carvalho, Maria Sameiro-Faria, Alice Santos-Silva, Luís Belo

**Affiliations:** 1 UCIBIO i4HB, Faculdade de Farmácia, Universidade do Porto, Rua Jorge Viterbo Ferreira 228, 4050-313 Porto, Portugal; up202210035@edu.ff.up.pt (S.S.); irina.filipa@hotmail.com (I.L.); mariasameirofaria@gmail.com (M.S.-F.); assilva@ff.up.pt (A.S.-S.); 2 FP-I3ID, FP-BHS, Universidade Fernando Pessoa, Praça de 9 de Abril 349, 4249-004 Porto, Portugal; mcarv@ufp.edu.pt; 3 LAQV/REQUIMTE, Faculdade de Farmácia, Universidade do Porto, Rua Jorge Viterbo Ferreira 228, 4050-313 Porto, Portugal; 4 RISE-Health, Faculdade de Ciências da Saúde, Universidade Fernando Pessoa, Fundação Ensino e Cultura Fernando Pessoa, Rua Carlos da Maia 296, 4200-150 Porto, Portugal; 5 Centro Hospitalar Universitário do Porto, Centro Materno-Infantil do Norte, Serviço de Pediatria, Unidade de Nefrologia Pediátrica, 4050-651 Porto, Portugal

**Keywords:** anemia, renal aging, elderly, chronic kidney disease, mechanisms

## Abstract

Renal aging is a physiological process characterized by structural and functional changes in the kidneys. The presence of disorders or pathologies can exacerbate these age-related changes, potentially leading to organ dysfunction. Chronic kidney disease (CKD), a significant global public health issue, is particularly prevalent in the elderly and is often associated with the age-related decline in kidney function. Anemia is one of the most frequent complications of CKD and is also highly prevalent in the elderly. Mild anemia, often multifactorial, is the most common presentation. Understanding the mechanisms driving anemia in this population is crucial to ensure appropriate treatment. The primary etiologies include nutritional deficiency, anemia of unknown cause, and anemia of chronic diseases, including CKD. This review provides an in-depth exploration of the complex pathophysiological mechanisms underlying anemia in elderly patients with CKD.

## 1. Introduction

The kidneys play a crucial role in the body’s homeostasis, namely in the regulation of water and electrolytes. This is possible due to their ability to filter blood and form urine with different concentrations of water and waste products. In addition, the kidneys perform endocrine functions, by producing renin, calcitriol and erythropoietin (EPO), which are responsible for regulating blood pressure, bone metabolism and producing red blood cells, respectively [1,2].

Advancing age is associated with changes in the structure and function of the kidneys, which may be exacerbated when associated with risk factors for renal diseases [3]. Certain pathologies, such as diabetes and arterial hypertension, promote the renal aging process, predisposing the elderly to chronic kidney disease (CKD), a condition that is becoming increasingly prevalent. CKD is associated with several complications, such as anemia, mainly caused by decreased red blood cell production and survival and/or iron deficiency. CKD-related anemia is usually associated with more severe disease stages and with an increased risk of cardiovascular events and mortality [4].

Since CKD is considered a global public health problem and CKD-related anemia is a significant complication associated with disease progression, early diagnosis is essential to implement interventions or therapies to slow disease progression, improve asthenia, cognitive and cardiac functions and, consequently, enhance the patients’ quality of life [5]. This is particularly challenging in elderly patients, as the signs and symptoms of CKD may overlap with physiological modifications that accompany aging. Thus, it is vital to understand the renal aging process and its progression to CKD, as well as the pathophysiology of anemia in the elderly patient with CKD.

This narrative review aims to synthesize existing evidence on the mechanisms of anemia in elderly patients with CKD. A comprehensive literature search was conducted using the PubMed database to identify relevant studies. The search strategy included the following keywords: “anemia”, “renal aging”, “elderly”, “chronic kidney disease”, and “mechanisms”. These terms were combined using the Boolean operators *AND* and *OR* to refine the search and maximize the retrieval of pertinent studies. No restrictions were applied regarding publication date or language to ensure a broad and inclusive selection of literature. Titles and abstracts of the retrieved articles were screened for relevance, and full-text articles were reviewed to extract key findings related to the pathophysiology and contributing factors of anemia in this patient population. Given the narrative review design, no formal quality assessment or meta-analysis was performed.

## 2. Aging Kidney

Renal aging is a physiological, not pathological, process that occurs with advancing age [6]. Although limited, healthy renal aging still allows for the maintenance of homeostatic balance under healthy conditions. However, the association of aging with risk factors for renal disturbances or chronic diseases, such as arterial hypertension, diabetes and obesity, can exacerbate these age-related changes [3].

**Figure 1:**
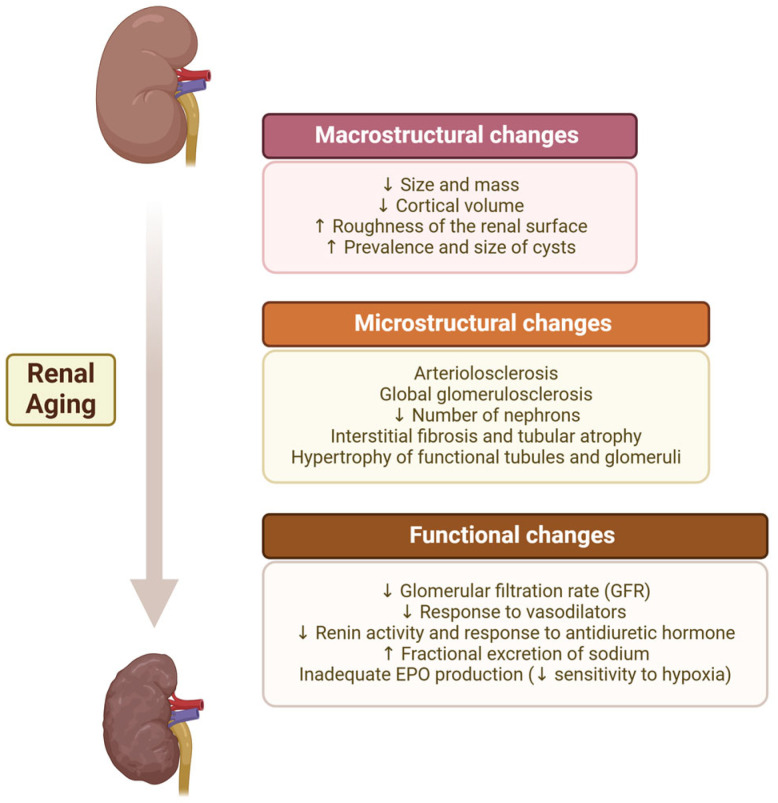
Anatomo-functional changes in the renal aging process. EPO, erythropoietin. Upper arrow means increase; down arrow means decrease. Created in BioRender. Carvalho, M. (2025) https://BioRender.com/z12s369, accessed on 8 March 2025.

It is important to recognize a normal physiological aging process in order to adopt an appropriate clinical approach [7]. The changes that the kidneys undergo with age are divided into two categories: anatomical changes (subdivided into microstructural and macrostructural) and functional changes (Figure 1) [8,9].

### 2.1. Anatomical Changes

#### 2.1.1. Macrostructural Changes

From the age of 50, loss of renal mass and volume is documented, with renal cortical atrophy and increased renal medullary volume, leading to a decline in renal parenchyma volume [10]. In the renal medulla, the interstitial tissue increases with the onset of signs of fibrosis and consequent atrophy of the renal pyramids, especially after the age of 70. The renal surface also undergoes changes, becoming more granular and scarred.

The amount of sinus fat increases slightly with age and may occasionally account for up to 17% of the post-mortem kidney weight. Also, renal parenchymal cysts become more frequent, larger and denser with age (Figure 1). The age-related increased prevalence of cysts is associated with overweight, with the presence of hypertension and albuminuria, and is more commonly observed in males [8,11].

#### 2.1.2. Microstructural CHANGES

##### Nephrosclerosis

Nephrosclerosis is commonly associated with CKD, but may be present in healthy renal aging, with its prevalence progressively increasing with age. Arteriosclerosis and arteriolosclerosis refer to the hardening and thickening of the walls of arteries and arterioles, respectively, due to increased fibrous tissue and deposition of hyaline material in the innermost layer of the vessels, the intima. Arteriosclerosis can lead to ischemia of the nephrons, resulting in glomerulosclerosis and tubular atrophy.

##### Nephron Hypertrophy

Hypertrophy of the remaining nephrons is a compensatory mechanism in response to the decrease in functional glomeruli alongside an increase in sclerotic and hyaline glomeruli. Hypertrophy causes the glomeruli to move apart and the glomerular density in the renal cortex to decrease. In healthy aging, hypertrophy is also observed in the tubules, which increase in volume. This leads to a decreased glomerular density in the renal cortex and increased tubular volume [3,12].

##### Number of Nephrons

The average number of nephrons with non-sclerotic glomeruli decreases with healthy aging, which associates glomerulosclerosis and nephrosclerosis [3,13]. Sclerotic glomeruli can be completely reabsorbed or undergo changes, such as atrophy [14,15]. Thus, the actual loss of glomeruli with aging becomes difficult to quantify [16]. There are also other factors, not related to aging, that influence the loss of nephrons, such as the number of nephrons in the newborn and its birth weight [3,6,8,17]. A low birth weight is indicative of a low number of nephrons, which is compensated by an increase in the single nephron glomerular filtration rate (snGFR) and an increase in glomerular size, allowing for a stable overall glomerular filtration rate (GFR) [3,18].

##### Glomerular Filtration Rate per Nephron (snGFR)

Individual variability in nephron number, due to acquired or hereditary causes, complicates its functional assessment at the nephron level [19]. The progressive loss of functional glomeruli due to glomerulosclerosis triggers a compensatory response, resulting in increased snGFR and glomerular capillary hydraulic pressure in an attempt to preserve total GFR. This compensation, however, leads to increased tension in the glomerular capillary walls, causing hypertension, hyperfiltration and further damage to the remaining nephrons.

### 2.2. Functional Changes

#### 2.2.1. Physiological Decline in GFR

With healthy aging, GFR declines progressively, beginning around the age of 30–40 years at a rate of approximately 0.8 mL/min/1.73 m^2^/year [20]. This decline in GFR may be caused by decreased glomerular lobulation and glomerulosclerosis and, consequently, by a reduction in the surface area available for filtration. In addition, a reduced cardiac output and increased renal arteriolar resistance can also reduce filtration at the kidney level. The permeability of the glomerular filtration barrier in humans is only minimally altered with aging [6,21].

#### 2.2.2. Hormonal Changes

Nitric oxide is a vasodilator, and its production in the kidneys tends to decrease with aging. This reduction contributes to decreased renal plasma flow and sodium retention and heightens the risk of kidney damage [7]. One of the main changes observed is compromised endothelial vasodilation, caused by diminishing responses to vasodilators, such as nitric oxide, and increasing sensitivity to vasoconstrictors, such as angiotensin II.

The renal response in the elderly is reduced, reflected in lower plasma renin levels, which lead to reduced angiotensin II and aldosterone levels. This regulation is extremely important due to its ability to regulate vasoconstriction of the glomerular capillaries, but also to maintain renal homeostasis, through the induction of aldosterone and antidiuretic hormone (ADH) secretion [22]. As plasma concentrations of renin and aldosterone are reduced, fractional excretion of sodium increases, and urinary concentrating capacity declines in the elderly, due to the compromised response to ADH [23].

Erythropoietin (EPO) is produced almost exclusively by the kidneys and is responsible for regulating the production of red blood cells. In the elderly, a decrease in renal sensitivity to hypoxia, in erythropoiesis efficacy and in the concentration of hemoglobin (Hb) are observed. Despite an increased production of EPO, as a compensatory mechanism associated with aging, increased erythrocyte turnover or increased resistance to EPO also occur. Thus, although EPO may be increased, the response appears to be disproportional, leading to gradually lower Hb levels with aging, still within normal reference ranges [24,25].

## 3. Chronic Kidney Disease

### 3.1. Definition and Classification

Chronic kidney disease (CKD) is a global public health problem, defined as the presence of kidney damage or loss of kidney function for three or more months. Its prevalence is rising worldwide and is highly associated with an increased risk of cardiovascular morbidity and mortality, premature death, and decreased quality of life [26,27].

According to Kidney Disease Improving Global Outcomes (KDIGO), this pathology is classified according to the cause, GFR, and albuminuria categories, allowing patients to be categorized according to the disease severity and risk [28,29]. These two last parameters complement each other as prognostic indicators [26].

Albuminuria is divided into three categories and can be calculated by the albumin excretion rate (AER) in 24 h urine or by the albumin/creatinine ratio (ACR) in type II urine, preferably. The use of creatinine reflects a reference due to its constant excretion over 24 h, providing an estimate with less associated error [29,30,31].

Regarding GFR, it can be determined in two ways: the measured glomerular filtration rate (mGFR), and the estimated glomerular filtration rate (eGFR), via equations [32]. While both have associated errors, eGFR using creatinine concentration is widely used, since this biomarker is routinely available in basic metabolic panels. Cystatin C is added to creatinine-based GFR estimates if a more accurate measurement is required. On the other hand, mGFR is reserved for scenarios requiring maximum accuracy.

According to eGFR, CKD is classified in five stages [29]. However, the GFR value alone may be insufficient for diagnosis, especially in earlier stages where patients are frequently asymptomatic. Therefore, CKD is diagnosed if, for three or more months, the GFR is less than 60 mL/min/1.73 m^2^ or if greater GFR is accompanied by the presence of one or more markers of kidney injury (Table 1) [26,29,33].

Despite its often silent early progression, signs or symptoms such as foamy urine, changes in urination frequency, asthenia, nausea, loss of appetite, and weight loss may be observed. In more advanced stages of the disease, other symptoms may appear, such as difficulty concentrating, paresthesia, edema, dyspnea, vomiting, insomnia, and halitosis (ammonia odor) [33].

### 3.2. Etiology and Risk Factors

CKD can be caused by primary kidney diseases. However, it is mainly caused by other diseases, such as diabetes, hypertension, systemic lupus erythematosus, human immunodeficiency virus (HIV) infection, chronic kidney infections, glomerular diseases such as glomerulonephritis, and polycystic kidney disease, among others [33,34].

CKD risk factors are divided into those that are modifiable and those that are non-modifiable (Table 2). When a potentially modifiable factor is identified, it should be corrected, as it will have an impact on the patient’s quality of life and the progression of CKD [35,36].

The RENA study assessed the prevalence of CKD in Portugal in a sample population with an average age of 56.7 years. The overall prevalence of CKD was 20.9%, and it was higher in individuals with diabetes mellitus than in those without (31.4% vs. 19.8%). Furthermore, it was found that individuals with normal weight, pre-obesity, and obesity of all classes presented CKD prevalences of 18.0%, 45.8% and 36%, respectively [37].

### 3.3. Prevalence in the Elderly

The prevalence of CKD is notably high among the elderly, which is mainly due to the increased frequency of risk factors for CKD, such as obesity, diabetes and hypertension, compounded by age-related renal function decline [38]. The third National Health and Nutrition Examination Survey (NHANES III) found that the prevalence of CKD was 8.5%, 12.6% and 39.4% in individuals aged 20–39 years, 40–59 years and 60 years or older, respectively [39].

Epidemiological studies have shown that decreased eGFR and increased albuminuria are common in the elderly [27,40]. However, it is important to understand whether these changes are consequences of healthy aging or whether they result from pathology [27,41].

### 3.4. Calculation of GFR in the Elderly

The increasing prevalence of CKD in older populations raises questions about the accuracy of eGFR measurements in this demographic. There are several factors that affect creatinine metabolism, namely age, gender, ethnicity, daily protein intake, malnutrition and medication [42]. These changes should be considered, as eGFR calculated for elderly individuals may be less accurate due to these factors [22,42].

To date, more than 50 equations have been proposed using creatinine to calculate eGFR. The first equation that was used worldwide was the Cockcroft–Gault (CG) equation [43]. However, it has some limitations, namely the fact that the group of individuals used for its development included few male individuals and it was created to estimate clearance creatinine rather than GFR [44].

In 1999, Levey, et al. proposed a new equation, the Modification of Diet in Renal Disease (MDRD) [45]. This equation is more complex than the CG equation and has undergone subsequent modifications. It has advantages over the CG equation, namely, the fact that it adds the ethnicity variable. However, it excludes individuals over the age of 70 years. Given that serum creatinine differs with age and between age groups, this equation tends to underestimate eGFR and, consequently, to overestimate the population prevalence of CKD [44].

In 2009, a new equation was developed by the Chronic Kidney Disease Epidemiology Collaboration (CKD-EPI) [46], which included patients over 65 years of age as well as diabetics. This is currently recommended by KDIGO, having undergone a change in 2021 to remove ethnicity as a variable. However, it still has limitations, namely the overestimation of eGFR in adults aged 18–30, as well as some differences in relation to ethnicity. On the other hand, the CKD-EPI equation that uses both markers (creatinine and cystatin C) has proven to improve accuracy for both ethnic groups, with smaller differences between these groups [44].

An equation was also later proposed by the Berlin Initiative Study (BIS) [47], which is limited to individuals over 70 years of age and which aimed to overcome the limitations of the CKD-EPI equation with regard to age and ethnicity [44].

Currently, the 2024 KDIGO guidelines recommend, in general cases, the use of the 2021 “CKD-EPI creatinine” [29,48] as the first choice, but the use of the “CKD-EPI creatinine-cystatin C” equation was also recommended as a confirmatory test, when necessary (Table 3) [49]. This guideline states that when abnormalities in creatinine metabolism are suspected, as is the case in the elderly, the initial test should include cystatin C. Furthermore, since the publication of KDIGO 2012, equations were developed by the European Kidney Function Consortium (EKFC), which obtained good results when compared with the CKD-EPI 2009 and 2021 equations [29,50].

### 3.5. Anemia—A Complication of CKD

CKD is associated with several complications, of different prevalence and severity, depending on the stage of the disease. However, these are interrelated and contribute to increased morbidity and mortality and reduced quality of life. The main complications of CKD include hypertension, cardiovascular complications, CKD-associated mineral and bone disorders, water and salt retention, metabolic acidosis, electrolyte disturbances, dyslipidemia, nutritional problems, and anemia [53,54].

A 2023 study conducted in the United States between 2016 and 2019 collected data from approximately five million individuals and assessed the relationship between anemia and eGFR. It was observed that the lower the eGFR, the higher the prevalence of anemia. Severe anemia was present in 1.3%, 3.1%, 7.5%, 17.4%, and 29.7% of men with eGFRs of 60–74, 45–59, 30–44, 15–29, and less than 15 mL/min/1.73 m^2^, respectively. In women, severe anemia was present in 1.9%, 3.9%, 8.6%, 19.4%, and 37.6%, respectively, in the same eGFR categories mentioned above [55].

Another population-based study involving more than 3000 individuals aged over 49 years (mean age 65 years) concluded that persistently elevated serum creatinine levels due to decreased renal function were associated with a higher prevalence of anemia in both genders. Based on World Health Organization (WHO) criteria, anemia was diagnosed in 1.6% of individuals with serum creatinine < 125 μmol/L, in 8% with serum creatinine between 150 and 174 μmol/L, and in 40% of patients with serum creatinine ≥ 200 μmol/L [56].

According to the KDIGO 2024 Clinical Practice Guidelines [29], adults with CKD with stage G3 and A1 diabetes and non-diabetics had a prevalence of anemia of 14.9% and 11.5%, respectively. This prevalence increases to 60.7% and 57.4%, respectively, in CKD stages G5 and A3, demonstrating the important increase in the prevalence of anemia with worsening CKD.

Both aging and CKD are undoubtedly contributors to anemia in the elderly. According to NHANES data, CKD anemia is likely to develop once the GFR is < 60 mL/min/1.73 m^2^ [57] and its prevalence is age-independent when eGFR < 45 mL/min/1.73 m^2^ [58]. Thus, an eGFR < 45 mL/min/1.73 m^2^ seems to be essentially permissive for anemia in CKD.

## 4. CKD Anemia

### 4.1. Multifactorial Nature

**Figure 2:**
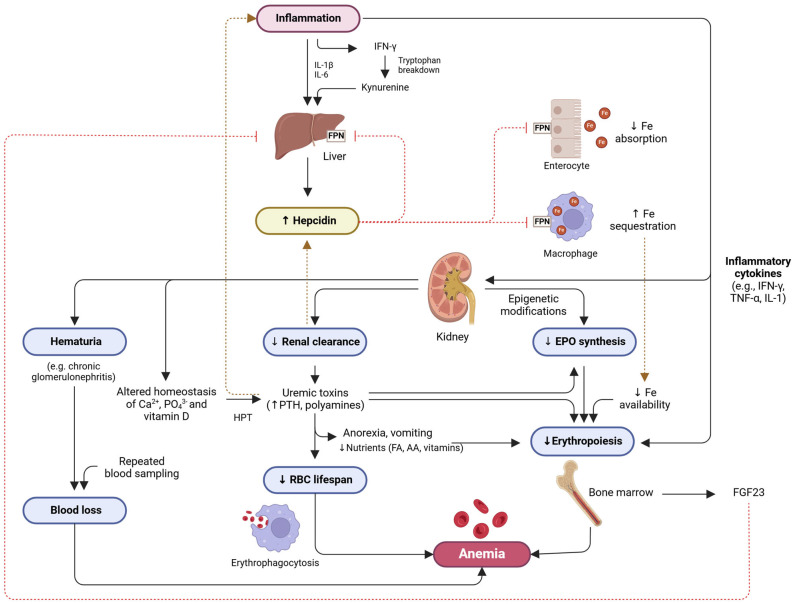
Mechanisms involved in anemia of chronic kidney disease (CKD). CKD is a long-standing progressive condition characterized by the deterioration of renal function, including decreased glomerular filtration rate (GFR) and compromised erythropoietin (EPO) production. CKD-related anemia is mainly attributable to inappropriate erythropoietin synthesis, particularly in advanced stages of the disease. Inflammation also plays an important role in promoting anemia, as CKD pathophysiology involves a final common activation of inflammatory pathways, irrespective of etiology. CKD and its associated risk factors (e.g., obesity) cause high levels of inteleukin-6 (IL-6), IL-1β, and interferon-γ (IFN-γ) that promote hepatic synthesis of hepcidin, a major peptide that regulates systemic iron metabolism. By interacting with its receptor ferroportin, a transmembrane iron-export protein, hepcidin inhibits iron absorption from enterocytes and its mobilization from macrophages of the reticuloendothelial system and from hepatic stores. Decreased renal hepcidin clearance further augments its circulating levels, aggravating iron availability for erythropoiesis. Inflammatory cytokines—such as IFN-γ and TNF-α—as well as uremic toxins (e.g., polyamines) inhibit bone marrow erythropoiesis. Although the exact mechanisms of uremic inhibition remain unknown, it is accepted that accumulation of uremic toxins in blood impairs erythropoietin synthesis. In addition, the uremic and pro-inflammatory systemic environment damages red blood cells (RBC), shortening their lifespan and further contributing to anemia. The uremic syndrome also leads to neurological symptoms (anorexia, nausea and vomiting) that may compromise adequate nutrient intake, limiting the availability of vital elements for erythropoiesis. Finally, secondary hyperparathyroidism is a common complication of CKD, and excessive levels of parathyroid hormone (a “uremic toxin”) hamper normal erythropoiesis by downregulating the erythropoietin receptors and cause a disturbed calcium metabolism in the peripheral RBC, promoting a hemolytic effect on these cells. AA, amino acids; EPO, erythropoietin; FA, fatty acids; FGF23, fibroblast growth factor-23; FPN, ferroportin; HPT, hyperparathyroidism; IFN, interferon; IL, interleukin; PTH, parathyroid hormone; TNF, tumor necrosis factor. Created in BioRender. Carvalho, M. (2025). https://BioRender.com/w32g215, accessed on 8 March 2025.

CKD anemia is mainly due to decreased production of erythropoietin by the kidneys, leading to impaired erythropoiesis, as well as shortened erythrocyte survival [4]. This type of anemia is predominantly normochromic, normocytic and hypoproliferative, usually occurring in more advanced disease stages. In cases of iron deficiency, also common in CKD, microcytic anemia may also be common, further accentuating the decline in Hb concentration [4,59]. Other causes that can exacerbate CKD anemia include blood loss, inflammation, and other nutritional deficiencies in addition to iron deficiency (Figure 2) [60,61].

### 4.2. Normal Erythropoiesis Versus Pathophysiology of CKD Anemia

Erythropoiesis is a complex physiological process regulated by EPO. This hormone, produced by peritubular interstitial cells in the cortex and outer layer of the renal medulla, stimulates erythroid cells in the bone marrow to proliferate and differentiate. In its absence, progenitor cells undergo apoptosis [62,63].

EPO synthesis is regulated by hypoxia inducible factor (HIF), a transcription factor that promotes erythropoiesis, not only by promoting EPO production by the kidneys, but also by improving iron absorption and utilization [64]. Under normoxic conditions, the presence of oxygen allows the activation of the prolyl-hydroxylase domain (PHD) enzyme. PHD hydroxylates proline residues of HIF-1α. The von Hippel–Lindau protein (pVHL) then recognizes the hydroxylated HIF-1α, binds to it and promotes its ubiquitination. The ubiquitinated HIF-1α is directed to the proteasome, where it is degraded. Thus, without the formation of the HIF complex, there is a blockage of the transcription of genes regulated by it, including the EPO gene. Under hypoxic conditions, PHD activity is inhibited, and HIF is not hydroxylated, allowing the HIF complex to enter the nucleus and to bind to the hypoxia response element (HRE), activating EPO gene transcription, while suppressing the hepcidin gene.

The major extrarenal source of EPO is the liver. However, the mechanism of hepatic EPO gene expression is different from that of the kidney, since it is much less sensitive to hypoxic stimuli [65,66,67]. In patients with CKD, reduced renal blood flow disrupts oxygen delivery to the kidney. Thus, the renal tissue needs to adapt to a lower oxygen consumption, considering the new normal tissue oxygen gradient. Consequently, PHD remains active, preventing the formation of the HIF heterodimer, and the EPO gene is not activated. Furthermore, since CKD is an inflammatory condition, there is production of inflammatory cytokines, including IL-1α, IL-1β, TGF-β and TNF-α, which inhibit EPO production.

Iron homeostasis is ensured by a process that regulates the absorption and secretion of iron, to prevent anemia or hemochromatosis. Since the body only absorbs a very small fraction of daily iron from diet,, most of the iron used in erythropoiesis comes from recycling of iron from senescent erythrocytes that have been phagocytosed by specialized reticuloendothelial macrophages. Thus, when serum iron levels are decreased, iron absorption in the gastrointestinal tract increases, but only within certain limits (increase up to 20%), since its absorption is mediated by receptors; in this case, the mobilization of iron from macrophages also increases. On the contrary, when serum iron levels are increased, iron absorption from the gastrointestinal tract as well as iron mobilization from macrophages decreases [64]. Since iron is an essential component of the heme group present in Hb, iron deficiency results in decreased Hb synthesis and consequently in hypochromia and microcytosis of erythrocytes. Low Hb levels induce hypoxia, promoting the stimulation of EPO production by the kidney peritubular cells and increasing erythropoiesis. However, if iron deficiency persists, deficient erythropoiesis persists [4,64].

Hepcidin is a peptide, produced by hepatocytes and freely filtered by the glomeruli, that can be measured in urine. The function of this peptide is to bind to ferroportin (an iron-exporting protein), forming a hepcidin–ferroportin complex. This complex is internalized and subsequently degraded in the lysosomes. Consequently, the release of iron into the bloodstream decreases, as does the release of iron by macrophages. When hepcidin levels are low, ferroportin develops a higher-than-normal activity, resulting in increased absorption of dietary iron in the intestine [68,69]. If dysregulated, this mechanism can result in hemochromatosis [70]. When hepcidin is increased, ferroportin activity is suppressed, leading to anemia, common in cases of inflammation, kidney failure and chronic diseases [64]. In fact, under inflammatory conditions and chronic diseases, such as CKD, there is an increased production of cytokines, such as IL-1, IL-6, TNF-α and interferon-γ, that stimulate the production of hepcidin and reduce the production of EPO and erythropoiesis [4,65,71].

Given the importance of hepcidin in the pathophysiology of inflammation-induced anemia, the scientific community has given considerable attention to regulators of *HAMP* gene expression, besides the well-known IL-6 stimulator via the JAK2/STAT3 signaling pathway [72,73]. It was recently demonstrated that fibroblast growth factor-23 (FGF23), a regulator of phosphate and mineral metabolism, with phosphaturic activity, decreases hepcidin expression induced by bone morphogenetic protein 6 (BMP-6) or IL-6 [74]. During inflammation, bone is the major source of C-terminal FGF23 cleaved peptides that reduce BMP-induced hepcidin secretion in the liver [75]. In the opposite sense, it was demonstrated that IL-1β activates hepcidin transcription via the CCAAT enhancer binding protein (C/EBP)-binding site in the HAMP promoter site [76].

Recent studies have also proposed a new mechanism for anemia of inflammation involving tryptophan metabolism. IFN-γ induces tryptophan breakdown via the kynurenine pathway [77], and lower tryptophan levels have been associated with decreased hemoglobin concentrations in anemia of chronic disease [78]. Kynurenine, the first of many bioactive metabolites of tryptophan, increases hepcidin expression and decreases EPO production in HepG2 cells by activating the aryl hydrocarbon receptor (AHR), which competes with hypoxia-inducible factor 2α [79].

Mechanisms of anemia involving epigenetic factors have also been proposed. For instance, LPS-induced inflammation increases circulating levels of microRNA 122 (MIR122), which reduces EPO expression in the kidney, suppressing erythropoiesis [80]. DNA hypermethylation of the EPO gene promoter in kidney cells can also suppress gene expression, reducing EPO levels [81,82]. Additionally, histone modifications may alter chromatin structure, further inhibiting EPO production [83]. Finally, epigenetic factors can also alter expression of iron-related genes. For instance, DNA methylation and histone acetylation can influence the expression of hepcidin [84,85].

The mechanisms that lead to anemia in CKD are therefore multifactorial. As shown in Figure 2, the pathophysiology of CKD involves the progressive reduction in EPO production, the reduced bone marrow response to EPO stimuli (due to uremic toxins and erythropoiesis-suppressing cytokines), and the decreased erythrocyte half-life. In addition, the increased production of hepcidin due to systemic inflammation resulting from CKD leads to ineffective use of iron stores, resulting in absolute or functional iron deficiency, which can be severe due to iron malabsorption. Blood loss, in cases of hematuria or repeated blood sampling for analytical purposes, may further aggravate CKD anemia.

### 4.3. Diagnosis

In patients with CKD, it is common to develop progressive and debilitating anemia which, if left untreated, can lead to dependence on blood transfusions [86]. According to NHANES [57], Hb concentration remains normal during the early stages of renal failure but inevitably decreases with the decline in eGFR and worsening of CKD. The clinical manifestations associated with anemia in CKD are common to any other anemia of different causes, so the observed symptoms of fatigue, thoracalgia, tachycardia, dyspnea and changes in sleep pattern are nonspecific for CKD anemia [4]. It is very important to determine other causes of anemia before starting any treatment, as some of these causes can be corrected, such as iron, vitamin B12 or folate deficiencies, hypothyroidism, hemolysis, blood loss, bone marrow disorders and hematological neoplasms [57,86].

According to the KDIGO Clinical Practice Guideline for Anemia in Chronic Kidney Disease [87], assessment of anemia in patients with CKD, regardless of age and disease stage, should include: hemogram (erythrocyte count, Hb concentration, hematocrit, hematimetric indices (mean corpuscular volume (MCV), mean hemoglobin concentration (MHC) and mean corpuscular hemoglobin concentration (MCHC)), leukocyte and platelet counts); reticulocyte count and reticulocyte maturity indices; serum iron, ferritin and transferrin saturation (TSAT); and serum levels of folate and vitamin B12.

Hb concentration is the most recommended parameter for diagnosing anemia. The reticulocyte count helps assess bone marrow activity, while red cell indices, such as MCV, MCH, MCHC and red blood cell distribution width (RDW), provide insights into anemia type. CKD anemia is often normochromic, normocytic and hypoproliferative, as in other chronic diseases. Cases of hereditary disorders of Hb formation, such as thalassemia or sickle cell anemia, generally present microcytosis, as is the case in cases of iron deficiency anemia. Hypochromia is also common in this type of anemia. Anemia due to vitamin B12 and folate deficiency, on the other hand, is generally macrocytic, as occurs in hematopoietic disorders, resulting from the action of toxins, or is associated with hypothyroidism, liver disease, and severe hemorrhages, and in the case of myelodysplasia, in which macrocytosis is associated with leukopenia or thrombocytopenia [87]. As regards MCH and MCHC, hypochromia is indicative of prolonged iron deficiency or hemoglobinopathy, such as thalassemia or sickle cell anemia. On the contrary, hyperchromia may be related to hemolytic uremic syndrome. Additionally, performing a peripheral blood smear allows for manual examination of cellular morphology and identification of potential abnormalities [87,88].

As mentioned, the reticulocyte count allows the assessment of bone marrow response to anemia. Decreased reticulocyte counts are indicative of low erythropoietic activity in the bone marrow (hypoproliferative erythropoiesis), which may be the result of low levels of EPO in circulation or may be due to bone marrow suppression by cytokines or other components resulting from the inflammatory process. Reticulocytes may be elevated in cases of bleeding and hemolysis [87].

The study of iron metabolism is used to assess iron storage and iron availability for erythropoiesis, by assessing ferritin and transferrin saturation (TSAT), respectively [86]. However, it is considered that these two parameters do not allow an accurate assessment of iron storage in the bone marrow [86,87].

Decreased ferritin (≤100 ng/mL) and TSAT (≤20%) levels are indicative of iron deficiency, suggesting a deficiency in iron availability for erythropoiesis. In chronic diseases, such as CKD, the levels of iron stored in bone marrow macrophages may be normal, but unable to be released due to the action of hepcidin. Ferritin is an inflammatory marker, so its interpretation should be performed with caution in patients with CKD, since inflammation is present in these patients [87].

### 4.4. Application of Artificial Intelligence in Managing Patients with CKD-Anemia

Artificial Intelligence (AI) plays a crucial role in the management of anemia in patients with CKD by enhancing diagnosis, treatment optimization, and patient monitoring. AI-powered predictive models can analyze patient data (e.g., hemoglobin levels, iron status, EPO levels) to diagnose anemia and predict disease progression [89].

Machine learning algorithms can suggest personalized intervention in CKD-related complications [90]. AI can help clinicians select the best therapeutic strategy, determining the optimal dosage of erythropoiesis-stimulating agents (ESAs) and iron supplementation, reducing the risk of over- or under-treatment [91]. These AI-based clinical decision support systems are already implemented in anemia treatment of hemodialysis patients [92]. AI-driven simulations can also help to predict the long-term outcomes of different anemia treatment strategies in CKD patients. Finally, AI-powered chatbots and virtual assistants provide patient education on anemia management and medication adherence.

## 5. Anemia in the Elderly

### 5.1. Definition

Anemia is highly prevalent in the elderly and often undervalued [93,94]. Although Hb levels naturally decline with age, anemia is not considered a normal finding in aged individuals.

The optimal Hb concentration values appropriate to physiological needs vary according to age, sex, altitude of residence, and specific conditions such as pregnancy. The WHO defines anemia according to Hb concentration: <13 g/dL in men and <12 g/dL in women [95,96]. However, the reference population used for this determination did not include individuals aged 65 and older, raising the question of whether this criterion applies to the elderly. This WHO definition also did not differentiate reference values between pre- and post-menopausal women [95,96,97]. New studies have been carried out to assess the prevalence of anemia in the elderly population, proposing new definitions of anemia for these individuals [98,99,100].

### 5.2. Adverse Health Effects

Although anemia in the elderly is predominantly mild, it is associated with several adverse effects, including decreased physical performance, frailty, reduced muscle strength (and, consequently, increased risk of falls), decreased cognitive performance, dementia, and increased risk and length of hospitalization. A well-described inverse relationship exists between Hb concentration and muscle strength, physical performance, disability, and mortality [95,101].

Anemia in the elderly is associated with a series of signs and symptoms associated with the frailty of the elderly, including weight loss, impaired mobility, generalized weakness, lack of balance and greater vulnerability to stress, collectively defining it as a geriatric syndrome [93,102].

### 5.3. Prevalence

As mentioned, the prevalence of anemia varies according to several factors, such as age, gender and ethnicity.

Regarding the association between anemia and age, the InCHANTI study [94] found a gradual decline in both Hb concentration and renal function with age in both genders, with the increased prevalence of anemia becoming progressively more evident with worsening renal function. Another study also carried out in Italy [101], involving approximately 8000 elderly individuals, confirmed this trend. It was observed that, among those aged 85 to 89, the prevalence of mild, moderate and severe anemia was 22.1%, 4.2% and 0.3%, respectively, and that in individuals aged 90 or over, the prevalence values were 31.5%, 9.0% and 1.3%, respectively. Based on these data, the study concluded that there is a marked increase in the prevalence of the various degrees of anemia severity at more advanced ages. This study also found that the prevalence of moderate anemia in hospitalized cases (13.5%) was significantly higher than that observed in non-hospitalized cases (1.6%) [101].

Other studies have also corroborated this trend, with more pronounced increases in older age groups [94,103,104]. A study from a New Zealand population [105] observed a prevalence of anemia in individuals over 65 years of age of 48%, in which 68% of these had mild to moderate anemia.

Regarding the prevalence of anemia according to gender, it is recognized that anemia in the elderly affects more men than women. According to the NHANES III, the prevalence of anemia is higher in women up to the age of 75. From the age of 75 to 84, there is a difference of approximately 5 percentage points between the two genders, with men having a higher prevalence; after the age of 85, the relationship reverses again, with women having a higher prevalence [96,106,107]. These data were also confirmed by the InCHANTI study [94]. As men age, they experience a decline in androgen levels, reducing their stimulating action on erythropoiesis [108,109]. The reduction in the prevalence of anemia in postmenopausal women may be related to the absence of menstruation. Due to these physiological changes between the sexes, at advanced ages, the reference values for the diagnosis of anemia may not adequately reflect the biological concept of normality for elderly people [107].

The prevalence of anemia also varies with ethnicity, with black individuals having a prevalence of anemia two to three times higher than that of Caucasians. A study conducted in the United States in elderly individuals (≥65 years old) found that the prevalence of anemia in non-Hispanic black men and women was 27.5% and 28.0%, respectively, and in non-Hispanic white men and women of similar ages, 9.2% and 8.7%, respectively. For Mexican–American men and women, the prevalence of anemia was 11.5% and 9.3%, respectively. However, the definition of anemia for different ethnicities is slightly different; a study from NHANES III showed that WHO-defined anemia is a strong predictor of mortality and mobility impairment among white and Mexican–American individuals, but that these criteria do not apply to black individuals [104,107].

### 5.4. Etiology

The etiology of anemia in the elderly can be divided into three main categories: nutritional deficiencies (mainly due to deficiency in iron, folic acid and vitamin B12); chronic diseases (such as CKD, inflammatory or infectious pathologies and tumors); and unknown causes [101,104,110,111].

In a study carried out in individuals aged ≥65 years, the etiology of anemia was distributed as follows: 56.6% due to nutritional deficiency, 21.1% due to hematologic neoplasia, 20.8% due to anemia of chronic diseases and 9.7% due to anemia of unknown cause [112].

The NHANES III study also examined the etiology of anemia in the elderly. For the diagnosis of nutritional deficiency anemia, the following were considered: the values of iron, vitamin B12 and folate. Anemia due to chronic diseases was diagnosed when serum iron was decreased without evidence of iron deficiency. Anemia due to CKD was considered when eGFR < 30 mL/min, and anemia due to unknown causes resulted from the exclusion of the remaining ones. It was reported that anemia due to nutritional deficiency, chronic diseases and due to unknown causes had prevalences of 34.3%, 32.2% and 33.6%, respectively [104].

**Figure 3:**
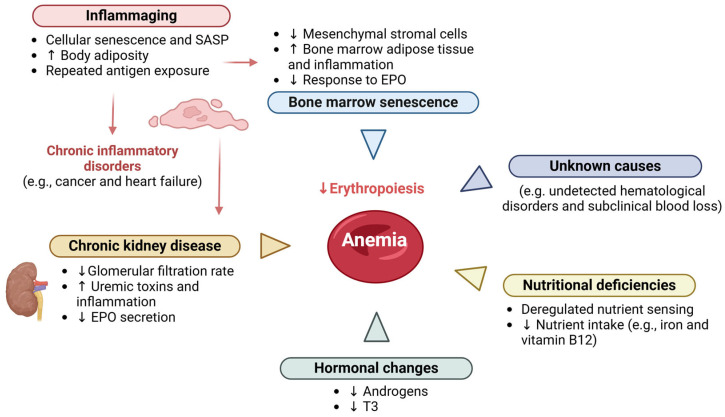
Etiology of anemia in the elderly. The etiology of anemia in the elderly is frequently multifactorial. Inflammaging is a low-grade chronic inflammation state that develops with advancing age and is a significant risk factor for multiple diseases that are highly prevalent in older individuals, such as type 2 diabetes, atherosclerosis and cancer. Inflammaging and/or chronic inflammatory disorders increase the synthesis of erythropoiesis-inhibiting cytokines, such as IFN-gamma and TNF-alpha. Decreased nutritional intake in the elderly may lead to deficiencies in nutrients that are vital for red blood cell production, namely iron, folate and vitamin B12. The physiological decline in renal function with age, together with aged-related diseases, such as diabetes and arterial hypertension, predispose the elderly patients to chronic kidney disease (CKD) development. In turn, CKD is characterized by reduced EPO synthesis, impairing the EPO-mediated prevention of apoptosis of erythroid progenitors in bone marrow. Other important hormonal changes, such as a decrease in androgen levels, may also contribute to decreased erythropoiesis. Finally, the aged bone marrow, with increased adipose tissue and an inflammatory ambient, becomes less responsive to EPO stimulation, exacerbating anemia in the elderly. EPO, erythropoietin; SASP, senescence-associated secretory phenotype. Created in BioRender. Carvalho, M. (2025). https://BioRender.com/a48x735, accessed on 8 March 2025.

The categorization of the etiology of anemia in the elderly has limitations, namely the fact that many cases considered unexplained are simply the exclusion of the other two categories. In fact, anemia in the elderly is often multifactorial (Figure 3) and derived from comorbidities that are common in older individuals (approximately 40% of individuals aged >80 years have four or more comorbidities) [113,114,115]. A study carried out on more than 19 thousand individuals aged ≥64 years, in Austria, concluded that multifactorial anemia was frequently observed. For example, the coexistence of renal failure (decreased eGFR) and increased inflammatory markers had a prevalence of 28.1% [114]. Determining the cause of anemia is extremely important to ensure that the most appropriate treatment is applied. However, the existence of comorbidities, as well as the use of multiple drugs, make diagnosis difficult in elderly patients.

#### 5.4.1. Nutritional Deficiency Anemia

The main cause of nutritional deficiency anemia in the elderly is iron deficiency, which typically presents with microcytosis. Diagnosis is essential to treat symptoms and identify the cause of iron deficiency, which is often related to pathologies of the gastrointestinal tract, such as gastritis, peptic ulcer, gastrointestinal polyps, cancer and inflammatory bowel disease [116]. Diagnostic tests include the evaluation of several parameters, such as serum ferritin, transferrin, serum iron, total iron-binding capacity and soluble transferrin receptor (sTfR), alongside measurements of hypochromia (mean blood cell volume, MCH) and microcytosis (mean cellular volume, MCV [116,117].

Although dietary supplementation has reduced folate deficiency, it still occurs due to malnutrition and alcoholism [118,119]. Vitamin B12 deficiency is mainly due to cobalamin malabsorption syndrome, to malabsorption in cases of atrophic gastritis, or therapeutic use of antacids. Unlike iron deficiency, anemia due to vitamin B12 and/or folate deficiency is macrocytic [116].

#### 5.4.2. Anemia Resulting from Chronic Diseases

Anemia of chronic diseases, which includes CKD, arises from acute or chronic infections, oncological diseases and chronic inflammatory diseases. This anemia is typically normocytic and normochromic, with mild to moderate reductions in Hb concentration. Hepcidin plays a crucial role in its pathophysiology, by inhibiting iron absorption on enterocytes, as well as iron release by macrophages, reducing the overall iron available for erythropoiesis. Diagnosis can be complicated by coexisting conditions, such as iron deficiency and thalassemia, which can result in microcytic anemia, but evaluation of iron metabolism parameters, such as transferrin, sTfR and serum ferritin, can aid in diagnosis [116,120]. However, the diagnosis can undergo evaluation of inflammatory markers, such as C-reactive protein, red blood cell sedimentation rate, interleukin-6 (IL-6) and hepcidin [116,121].

#### 5.4.3. Anemia of Unknown Cases

Anemia of unknown cause in the elderly is usually mild, normocytic and hyperproliferative. It is diagnosed when nutritional deficiencies, CKD or inflammatory conditions are excluded. This type of anemia may be associated with several age-related physiological mechanisms, such as declining renal function, androgen deficiency, chronic low-grade inflammation associated with aging (inflammaging), myelodysplastic syndromes and impaired response to EPO [113,122,123]. These patients commonly have lower EPO levels than in cases of iron deficiency, which suggests the presence of a subclinical pro-inflammatory state [122,124].

## 6. Conclusions

Renal aging is a natural physiological process that results in a progressive decline in kidney function. Distinguishing healthy renal aging from pathological conditions is essential for appropriate clinical management. CKD is considered a global public health problem, and its prevalence has increased over the years, significantly affecting the quality of life of patients, particularly the elderly.

Future research should address the identification of earlier and more specific markers (etiology-related) of CKD onset [125]. The analysis of big databases using machine learning and AI will allow us to better predict CKD onset/progression and anemia development, permitting earlier preventive strategies in high-risk patients. Further AI-driven simulations will also help predict the long-term outcomes of different anemia treatment strategies in CKD patients.

The scientific community must also be aware of the limitations of current analytical biomarkers in the evaluation of bodily iron stores, particularly in the presence of inflammation. For instance, ferritin and transferrin levels are influenced by inflammation; soluble transferrin receptor is independent of inflammation but is influenced by erythropoietic activity, limiting its utility in EPO-treated patients [126].

The development of new therapeutic approaches for renal anemia should also be addressed, and AI has the potential to accelerate the discovery of new treatments. Hepcidin antagonists have been developed, with few entering clinical trials [72]. The introduction of drugs that improve ESA effectiveness deserves special attention, as resistance to ESA usually drives an increment in its dosage, with potential associated risks, namely cardiovascular hazards (e.g., hypertension, thrombosis and cardiovascular mortality) [127]. Anti-inflammatory molecules may decrease EPO doses, as was observed with docohexanoic acid in subjects under hemodialysis [128]. Finally, understanding the epigenetic landscape of CKD-related anemia opens new avenues for treatment. Epigenetic drugs, such as DNA methylation inhibitors or histone modification modulators, could help restore normal gene expression and improve erythropoiesis. This may be particularly relevant in the elderly, as epigenetic alterations are a hallmark of aging.

## Figures and Tables

**Table 1:** Findings evidencing kidney injury.

Albuminuria (AER ≥ 30 mg/24 h or ACR ≥ 30 mg/g) Modifications in urinary sediment Changes in electrolytes or changes caused by tubular damage Anatomical or structural anomalies detected by imaging Pathological anomalies detected by histology History of kidney transplant

AER, albumin excretion rate; ACR, albumin/creatinine ratio.

**Table 2:** Risk factors for chronic kidney disease (CKD).

Non-Modifiable Risk Factors	Common Modifiable Risk Factors	Less Common Modifiable Risk Factors
▪ Advanced age ▪ Black race ▪ Family history of CKD or cardiovascular disease ▪ History of acute kidney injury ▪ Low birth weight	▪ Diabetes ▪ Hypertension ▪ Obesity ▪ Dyslipidemia ▪ Metabolic acidosis ▪ High protein diet ▪ Smoking	▪ Anemia ▪ Abusive use of drugs (e.g., AINE, certain antibiotics) ▪ Consumption of nephrotoxic plants ▪ Hyperuricemia ▪ Hypercalcemia ▪ Hyperphosphatemia

AINE, non-steroidal anti-inflammatory drugs; CKD, chronic kidney disease.

**Table 3:** CKD-EPI creatinine and CKD-EPI creatinine–cystatin C equations for calculating glomerular filtration rate.

**CKD-EPI Creatinine Equation (2021)**	eGFR_cr_ = 142 × min(*S_cr_*/*k*,1)*^α^* × max(*S_cr_*/*k*,1)^−1.200^ × 0.9938*^age^* × 1.012[*if female*]
**CKD-EPI Creatinine-Cystatin C** **Equation (2021)**	eGFR_cr−cys_ = 135 × min(*S_cr_*/*k*,1)*^α^* × max(*S_cr_*/*k*,1)^−0.544^ × min(*S_cys_*/0.8,1)^−0.323^ × max(*S_cys_*/0.8,1)^−0.778^ × 0.9961*^age^* × 0.963[*if female*]

eGFR_cr_—Estimated glomerular filtration rate from creatinine; eGFR_cr-cys_—Estimated glomerular filtration rate from creatinine and cystatin C; Scr—Standard serum creatinine (mg/dL); Scys—Standard serum cystatin (mg/dL); k = 0.7 (female) or 0.9 (male); α = −0.241 (female) or −0.302 (male); min (Scr/k,1) = minimum Scr/k or 1.0; max (Scr/k, 1) = maximum Scr/k or 1.0. Adapted from National Kidney Foundation (NKF) [51,52].
